# Characterization and Evaluation of Lactic Acid Bacteria from Feline Milk for Probiotic Properties

**DOI:** 10.3390/ani15131990

**Published:** 2025-07-07

**Authors:** Haohong Zheng, Jiali Wang, Yunjiang Liu, Zhijun Zhong, Haifeng Liu, Ziyao Zhou, Guangneng Peng

**Affiliations:** Key Laboratory of Animal Disease and Human Health of Sichuan Province, College of Veterinary Medicine, Sichuan Agricultural University, Chengdu 611130, China

**Keywords:** lactic acid bacteria, antimicrobial properties, feline milk, evaluation

## Abstract

Antibiotic overuse has contributed to the emergence of multidrug-resistant (MDR) bacteria, posing a serious public health threat. Pets may act as reservoirs of MDR bacteria, with the potential to transmit these pathogens to humans. This study aimed to identify probiotic alternatives to antibiotics by isolating and evaluating lactic acid bacteria (LAB) from feline milk. In addition to conventional in vitro assessments such as growth kinetics, adhesion ability, safety, and antipathogenic activity, this study also evaluated the antioxidant capacity and production of beneficial metabolites. Three LAB strains were isolated from feline milk, including two strains of *Lactobacillus plantarum* (M2 and M3) and one strain of *Weissella confusa* (M1). Resistance assays revealed that strains M2 and M3 exhibited high survival rates under stress conditions, including exposure to bile salts, acidic environments, artificial intestinal and gastric juice. Notably, strain M3 demonstrated strong auto-aggregation ability (73.39%) and high hydrophobicity toward trichloromethane (62.16%). It was also nonhemolytic and susceptible to various β-lactam antibiotics. Furthermore, strain M3 exhibited potent antimicrobial activity in both co-aggregation and Oxford cup assays. Overall, *L. plantarum* M3 displayed superior probiotic properties, suggesting its potential as an adjunct or alternative to antibiotics in managing MDR bacterial infections in cats.

## Simple Summary

Probiotics are beneficial bacteria that help maintain a healthy balance in the digestive system. They are often added to food or used as supplements to support gut health and immunity. In this study, we isolated and identified bacteria from the milk of healthy cats to explore their potential as probiotics. After testing their ability to survive harsh conditions like stomach acid and bile, and to fight harmful bacteria, several strains showed strong probiotic characteristics. These bacteria were also safe and showed potential to improve intestinal health. Our findings suggest that cat milk may be a valuable source of new probiotics, which could be developed into supplements or added to pet food to improve the health of cats and possibly other animals. This research provides a foundation for creating natural and effective ways to support animal health through diet.

## 1. Introduction

The discovery and application of antibiotics have long provided an effective means of treating bacterial infections [1]. However, numerous bacterial species had already developed resistance mechanisms long before the widespread production and clinical use of antibiotics [2,3]. Some of these have evolved into antimicrobial-resistant (AMR) strains or even “superbugs”—microorganisms that exhibit resistance to most known antimicrobial agents [4,5]. According to a report published in *The Lancet*, bacterial AMR was associated with approximately 5 million deaths globally in 2019, ranking as the third leading cause of death after cardiovascular diseases [6]. This growing public health crisis urgently demands global attention and effective countermeasures [7].

Due to heightened public health concerns and the enforcement of stringent veterinary pharmaceutical regulations, the development and availability of antibiotics for companion animals, such as cats, have lagged behind those for human use [8]. Consequently, treatment options for bacterial infections in pets are relatively limited, increasing the risk of AMR infections among these animals. Moreover, the close physical interactions between humans and their pets facilitate the potential zoonotic transmission of AMR pathogens [9,10], thus posing additional public health risks. Therefore, identifying safe and effective alternatives to antibiotics for use in companion animals has become an urgent priority.

Probiotics, defined as live microorganisms that confer health benefits to the host when administered in adequate amounts [11], have emerged as promising candidates to substitute for antibiotics. Their efficacy stems from multiple and targeted antibacterial mechanisms, as demonstrated in various studies [12,13,14]. Among these, milk-derived probiotics have garnered particular interest due to their high abundance, host compatibility, and excellent safety profiles [12,15].

Probiotics have already been applied in feline medicine for various purposes, including the prevention and treatment of acute gastroenteritis, management of inflammatory bowel disease (IBD), and reduction of allergic responses [16]. However, most of these probiotics are derived from non-feline sources such as plants or other animal species, introducing exogenous microorganisms that may disrupt the native gut microbiota of cats [17,18].

In this study, we selected feline milk as the source material for the isolation of potential probiotic strains. We comprehensively evaluated the isolates’ growth kinetics, adhesion capacity, antibacterial activity, and other relevant probiotic properties. Notably, this is the first study to assess the antioxidant potential and beneficial metabolite production of LAB strains isolated from feline milk. These findings aim to provide a theoretical foundation for the development of cat-specific probiotic strains as potential alternatives to antibiotics.

## 2. Materials and Methods

### 2.1. Sample Collection

Three feline milk samples were collected by a nationally licenced veterinarian in sterile tubes from three domesticated, healthy, and postpartum cats with their owners’ informed consent. Neither of the experimental animals had received antibiotics or probiotics for 60 days. The samples were promptly transported to the laboratory and held at 4 °C in the refrigerator until handling.

### 2.2. Isolation and Purification

#### 2.2.1. Isolation

To isolate suspicious lactic acid bacteria from feline milk, 1 mL of each sample was inoculated in the 10 mL centrifuge tubes with 4 mL MRS broth (Hopebio, Qingdao, China) and cultured in a shaking incubator at 37 °C until the mixture became muddy or had bacterial precipitation at the bottom of the tubes. After serial dilution, 100 μL of each serial diluent was inoculated in the MRS agar medium (Hopebio, Qingdao, China) by the method of plate smearing and the plates were transferred into a 37 °C shaking incubator for 24 to 72 h until well-isolated single colonies were observed. Only different and yellow colonies were selected as the potential strains and were subjected to the following purification experiment.

#### 2.2.2. Purification

The above suspicious LAB was inoculated in the MRS agar medium by the plate streak method and cultured in a 37 °C shaking incubator three times for purification. After being characterized as LAB by Gram-staining, morphology appraisal, catalase test, and coagulase reaction, according to previously standard procedures [15], the Gram-positive, bacilli and cocci, catalase- and coagulase-negative, and nonmotile isolates were selected, stored in 25% glycerol solution, and held at −80 °C in the refrigerator until species identification.

### 2.3. Species Identification

Each isolate of DNA was extracted using a commercial DNA kit (TIANGEN Biotech Co., Ltd., Beijing, China), and its quality was measured by an ultramicro-ultraviolet spectrophotometer (Thermo Scientific, Waltham, MA, USA). The general primer 27F(5′-AGGTTTTGATCCTGGCCAG-3′) and 1492R(5′-TACGACTTAACCCCAATCGC-3′) were used to amplify the partial 16S rDNA gene of all isolates. An aliquot of the PCR amplification product was loaded on a 1% agarose gel and visualized under UV light after staining with Ethidium Bromide. The rest of the products were sent to the Wuhan Qingke Biotechnology Co., Ltd. (Wuhan, China) for 16S rDNA sequencing. The sequencing results were matched with the existing gene sequences in NCBI GenBank by using Blast (https://blast.ncbi.nlm.nih.gov/Blast.cgi?PROGRAM=blastn&PAGE_TYPE=BlastSearch&LINK_LOC=blasthome, accessed on 9 June 2025). The phylogenetic tree was built by Mega 7.0 (Mega Limited, Auckland, New Zealand) using the maximum likelihood method.

### 2.4. Assessment of the Growth Kinetics

#### 2.4.1. Growth Curve

After being activated three times, three isolates were inoculated in the MRS broth following the volume ratio of 2% (*v*/*v*), and the total cultural volume was 1 mL. Then, a multifunctional enzyme marking instrument—Thermo Fisher Varioskan Flash (Thermo, Waltham, MA, USA)—was used to detect the initial OD_600_ values of the mixtures. Next, the inoculated medium was transferred to a 37 °C shaking incubator and cultured persistently. During the cultural time, the multifunctional enzyme marking instrument was used to detect the OD_600_ values of all isolates every 2 h for the first 24 h, and every 4 h for the next 24 h.

#### 2.4.2. Tolerance for Acid and Bile Salt

According to the study of Dowarah et al. [19], the acid tolerance was determined at acidic (2.0, 3.0, 4.0) pH by adjusting the pH of the medium by 1 M HCl (Hopebio, Qingdao China). Different bile salt (Solarbio, Beijing, China) concentrates (0.01%, 0.03%, 0.05%) were added to the broth to construct high-bile salt environments. A total of 180 μL of MRS broth in a regular environment and different pH levels or concentrates of bile salt were inoculated with 20 μL of overnight grown cultures in 96-well cell culture plates and incubated for 24 h at 37 °C. The values of OD_600_ of the negative control (A_con_) and the other mixture (A_mix_) were detected to calculate the livability (%) as (A_con_/A_mix_ × 100).

#### 2.4.3. Tolerance for Simulating the GIT Environment

According to the study of Zhang et al. [15], sterilized artificial gastric juice and intestinal juice were prepared for this assay. The bacterial cells were then resuspended in 10 mL of simulated gastric juice (0 h) and incubated at 37 °C for 3 h (3 h). The bacterial cells obtained by centrifugation of gastric juice were transferred again into 10 mL of simulated intestinal juice and incubated at 37 °C for 4 h (7 h). Finally, the plate-counting method was used to count the mixed bacterial solution at 0 h, 3 h, and 7 h, and the survival rates of isolates at different time points were calculated according to (N_test_/N_con_ × 100), where N_test_ represents the original bacterial count and N_con_ represents the bacterial count results at each time point.

### 2.5. Assessment of Adhesion Capacity

#### 2.5.1. Auto-Aggregation

For the auto-aggregation assay, the free cultures of three isolates were centrifuged at 4500 r/min for 10 min to collect the bacterial pellet, washed twice with PBS buffer, and adjusted to OD_600_ = 0.8 with the same buffer. After vortexing for 10 s, 4 mL of the bacterial suspension was incubated at 37 °C for 6 h. A multifunctional enzyme marking instrument was used to measure the absorbance (600 nm) at the beginning (A_con_)and the end (A_test_). The result of the auto-aggregation assay was presented by the co-aggregation ratio (%), which was calculated as 1 − (A_con_/A_test_) × 100.

#### 2.5.2. Hydrophobicity Assay

The bacterial suspension was obtained as described above, and 2 mL of them were mixed with 2 mL of ethyl acetate (CHRON, Chengdu, China), xylol (CHRON, Chengdu, China), or trichloromethane (CHRON, Chengdu, China), respectively, and the origin values of OD_600_ (A_con_) were measured. After vortexing for 10 min, they were set aside at 25 °C for 40 min until the stratified phenomenon occurred. The aqueous phases were collected carefully, and their values of OD_600_ (A_test_) were measured to calculate the cell surface hydrophobicity rate (%) as 1 − (A_con_/A_test_) × 100.

#### 2.5.3. Adhesion to Caco-2 Cell Line

To directly evaluate the adhesive ability of probiotics to intestinal epithelial cells, we co-cultured three isolates with Human Colon Adenocarcinoma cells (Caco-2 cells). According to a previous study [20], monolayer Caco-2 cells were cultured in high-glucose Dulbecco’s modified Eagle’s medium (DMEM) (Solarbio, Beijing, China), supplemented with 10% fetal bovine serum (FBS) (Sangon Biotech, Shanghai, China) under 5% CO_2_ atmosphere at 37 °C and detached from the flask with 2 mL of trypsin (Solarbio, Beijing, China) at 70% confluency. Caco-2 cells and three isolates were co-cultured as described in the study of Wang et al. [21]. After counting by a hemocytometer, cell suspensions were diluted to 1 × 10^5^ cells/mL (V_c_) concentration, and 1 mL of diluent was mixed with 1 mL of OD_600_ = 0.8 each LAB suspension in a 6-well cell culture plate. Subsequently, mixtures were incubated at 37 °C for 2 h, washed twice with PBS buffer, and lysed with trypsin. The lysates of Caco-2 cells were serially diluted, spread on MRS agar medium, and incubated at 37 °C for two days. The single colonies of isolates were counted (V) to calculate the adhesion index as V/V_c_.

### 2.6. Safety Assessment

#### 2.6.1. Hemolytic Capacity

Three isolates were inoculated in sheep blood plates (Hopebio, Qingdao, China) and incubated at 37 °C for two days to observe whether they generated hemolytic phenomenon. *Escherichia coli* (ATCC 25922), verified to produce a β-hemolytic ring by a previous study [22], was set as a positive control in this test.

#### 2.6.2. Antibiotic Susceptibility Assay

The antibiotic susceptibility of the selected LAB strains was assessed by the disc-diffusion test. Eighteen antimicrobials (Shunyoubio, Shanghai, China) were applied in this test, including penicillin G (P, 10 μg), ampicillin (AMP, 10 μg), amoxicillin (AML, 25 μg), erythromycin (E, 15 μg), Cefuroxime (CXM, 30 μg), cefotaxime (CTX, 30 μg), Oxacillin (OX, 5 μg), Cefazolin (KZ, 30 μg), Gentamicin (GM, 10 μg μg), Amikacin (AK, 30 μg), Kanamycin (KM, 5 μg), streptomycin (S, 10 μg), Norfloxacin (NOR, 5 μg), Rifampicin (RD, 5 μg), clindamycin (DA, 10 μg), chloramphenicol (C, 30 μg), tetracycline (TE, 30 μg), and vancomycin (VA, 30 μg). The fresh cultures of three isolates were diluted to OD600 = 0.8, and 100 μL of them was spread on MRS agar plates and dried thoroughly, preparing for the antibiotics disc-diffusion test. After different antibiotic discs were placed on them, the plates were transferred to a 37 °C incubator for two days. Antibiotic susceptibility was classified as resistant (R), moderately susceptible (M), or sensitive (S) based on the diameter of the zone of inhibition (mm) according to the parameters of the Clinical and Laboratory Standards Institute [23].

### 2.7. Assessment of Antipathogenic Activity

#### 2.7.1. Antimicrobial Ability

Intend to evaluate the antipathogenic capability of the cell-free supernatant (CFS) of three isolates, we detected their antagonistic activity against common intestinal pathogens, including *E. coli* (ATCC 25922), *Staphylococcus aureus* (ATCC 25923), *Salmonella braenderup* (H9812), and *Pseudomonas aeruginosa* (PAO 1), by the Oxford cup method. Referring to Zhang et al. [15], after three isolates were inoculated in the MRS broth at 37 °C for 24 h, underwent 4500 r/min centrifuging for 10 min, and were filtered through a 0.22 μm filter membrane, each CFS of three LAB was collected. At the same time, four pathogens were cultured following the same conditions, diluted to 10^7^ CFU/mL, and smeared 100 μL to the LB solid medium. Each Petri dish was placed in three sterile Oxford cups, and 200 μL CFS of isolates was added to the cups. After incubating at 37 °C for 24 h, all the diameters of the inhibition zone were measured and recorded. Referring to the previous study, the antipathogenic activities of these LAB were divided into four ranges: I, 8 mm < zone diameters ≤ 12 mm; II, 12 mm < zone diameters ≤ 16 mm; III, 16 mm < zone diameters ≤ 20 mm; and IV, 20 mm < zone diameters.

#### 2.7.2. Co-Aggregative Assay

The co-aggregative assay was carried out to assess the ability to gather and eliminate pathogens of three isolates, as described by Reuben et al. [12]. After mixing 2 mL of each LAB isolate and each pathogen culture, the mixture was vortexed and incubated for 2 h at 37 °C. In the experiment, 4 mL of each bacterial suspension was regarded as a control tube. The absorbance of each mixed suspension was then measured at 600 nm (A_mix_) and compared with those of the control tubes containing the LAB strain (Astrain) and the specific pathogen (A_pathogen_) at 2 h of incubation. Co-aggregation (%) was calculated as [1 − A_mix_/(A_strain_ + A_pathogen_)/2] × 100.

### 2.8. Antioxidative Capacity

The three isolates’ bacterial suspensions (BSs) or CFS were prepared in advance for antioxidative capacity assay. The CFS was collected by centrifuging overnight culture of isolates at 8000 r/min, 4 °C for 10 min. After washing twice with PBS buffer, the bacterial pellet was resuspended in PBS buffer, and it was adjusted to OD_600_ = 0.8, acquiring the BSs.

#### 2.8.1. Tolerance for H_2_O_2_

The tolerance for H_2_O_2_ was evaluated by detecting the livability of three isolates in MRS liquid mediums containing different concentrations of H_2_O_2_. Overnight cultures of LAB were inoculated into the MRS broth with 1.0 mmol/L, 1.5 mmol/L, and 2.0 mmol/L H_2_O_2_ (CHRON, Chengdu, China). The MRS broth inoculated isolates without adding H_2_O_2_ were set as the blank control (A_con_). After incubating at 37 °C for 8 h, OD_600_ of the cultures (A_text_) was measured to calculate the livability (%) as A_text_/A_con_ × 100.

#### 2.8.2. Total Antioxidant Capacity

Total Antioxidant Capacity Assay Kits with Rapid ABTS (Beytime Biotech Co., Shanghai, China) was applied to detect the antioxidant capacity of three isolates. Detailed operation steps are described by Hua et al. [24]. Trolox-Equivalent Antioxidant Capacity (TEAC) was used to represent the total antioxidant capacity of the three isolates.

#### 2.8.3. DPPH Radical Scavenging Ability

The 1,1-diphenyl-2-picrylhydrazyl (DPPH) radical scavenging ability of LAB was determined according to the method described in the literature [25]. To assess antioxidant activity, 2 mL of 0.2 mmol/L DPPH (Biotopped, Beijing, China) absolute ethanol solution was added to a centrifuge tube containing 1 mL of lactic acid bacteria cell-free supernatant or bacterial suspension. The mixture was vortexed and allowed to react for 30 min at room temperature in the dark. Subsequently, it was centrifuged at 8000 rpm for 10 min to collect the supernatant. The OD value of the supernatant was measured at a wavelength of 517 nm using a multifunctional enzyme marker instrument (A_text_). Anhydrous ethanol was used as the blank group instead of the DPPH absolute ethanol solution (A_blank_), and distilled water was used as the control group instead of the sample for the reaction (A_con_). The experiment was repeated three times, and subsequently, the DPPH free radical scavenging rate of LAB was calculated as [1 − (A_text_ − A_blank_)/A_con_] × 100.

#### 2.8.4. Reducing Capacity

The reducing capacity of three isolates was detected according to the method described by Wang et al. [21]. The OD_700_ of the reaction solution was measured to represent the reducing capacity of three isolates.

### 2.9. Metabolite Assessment

#### 2.9.1. Bile Salt Hydrolase (BSH) Production

The BSH production activity was determined by the Berthelot colorimetry method described by Fang et al. [26] with some modifications. Firstly, 20 μL of CFS was mixed with 10 mM dithiothreitol (Sangon Biotech, Shanghai, China) before ultrasonic crushing commenced for 10 min. After centrifuging at 8000× *g*, 4 °C for 10 min, the cell-free extract (CFE) was collected for the next step. Next, 10 μL of CFE or CFS was mixed with 180 μL of PBS buffer and 10 μL of 0.1 mol/L sodium taurocholate (Sangon Biotech, Shanghai, China). The mixture was vortexed and bathed at 37 °C for 30 min, and then 200 μL of 15% trichloroacetic acid (TCA) (CHRON, Chengdu, China) was added and reacted for 1 min. After centrifuging at 8000× *g*, 4 °C for 10 min, 100 μL of suspension was collected in the centrifuge tube and mixed with 1.9 mL ninhydrin solution (CHRON, Chengdu, China). The mixture was transferred into boiling water for 15 min and into ice water for 5 min, and the OD_570_ value was measured to calculate the concentration of BSH based on the glycine standard curve, which was constructed using glycine standards at concentrations of 0, 0.1, 0.2, 0.3, 0.4, and 0.5 μmol/L. One unit (U) of BSH produces 1 μg of glycine per minute at 37 °C.

#### 2.9.2. Extracellular Polymeric Substances (EPS) Production

The EPS production abilities of three isolates were evaluated according to the method described by Ren et al. [27]. The CFS was mixed with 800 mg/mL TCA to a final concentration of 40 mg/mL and set at 4 °C overnight. After centrifuging at 8500 r/min, 4 °C for 10 min, 250 μL of CFS was mixed with 250 μL of 6% (*w*/*v*) phenol solution (CHRON, Chengdu, China) and 1 mL of concentrated sulfuric acid (CHRON, Chengdu, China) and cooled at 25 °C. The OD_490_ value of the reaction solution was measured, and glucose with concentrations of 3.125, 6.25, 12.5, 25, 50, and 100 mg/L was used to make a standard curve to calculate the concentration of EPS.

#### 2.9.3. γ-aminobutyric Acid (GABA) Production

Referring to the method of Berthelot colorimetry described by Wang et al. [21], three isolates were inoculated in L-glutamic acid liquid medium, which was prepared by adding 10 g/L L-glutamic acid (CHRON, Chengdu, China) in MRS broth and adjusting pH to 6.3 at 37 °C for 48 h to obtain the fermentation broth (FB). The 600 μL supernatant of fermentation medium was obtained by centrifuging at 16,000× *g* for 15 min. Subsequently, the supernatant was mixed with 2 mL of 0.1 mol/L sodium tetraborate solution (CHRON, Chengdu, China), 800 μL of 6% phenol (*v*/*v*), and 900 μL of 10% sodium hypochlorite (CHRON, Chengdu, China) (*v*/*v*) and vortexed for 10 s. After being transferred into boiling water for 10 min and into ice water for 20 min, 4 mL of 60% ethanol solution was added and mixed evenly. The OD_645_ value of the mixture was measured to calculate the concentration of GABA according to the GABA standard curve, which was constructed using GABA standards at concentrations of 0, 0.2, 0.4, 0.6, 0.8, and 1.0 g/L.

### 2.10. Statistical Analysis

The results were shown as mean ± standard deviation (SD) and analyzed by the SPSS 27.0 statistical package (International Business Machines Corporation, Armonk, NY, USA). The figures were generated using GraphPad Prism 9.5 (GraphPad Software Inc., San Diego, CA, USA).

## 3. Results

### 3.1. Species Identification

**Figure 1:**
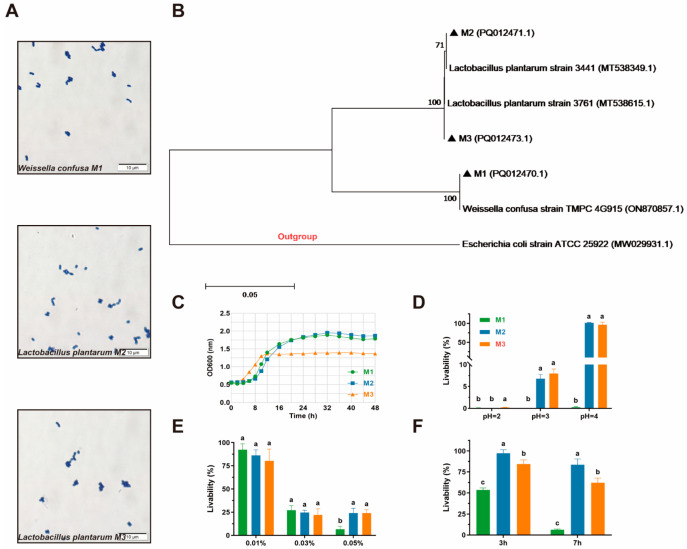
The results of growth kinetics and resist capacity assessment. All the results are represented as the mean ± SD. (**A**) Gram-stain microscope image of three isolates; (**B**) the phylogenetic tree based on 16S rDNA genes of the type strains and three isolates derived from feline milk; (**C**) growth curve of three isolates; (**D**) livability of three isolates in different acidic environments; (**E**) livability of three isolates in different concentrations of bile salt; (**F**) livability of three isolates in artificial gastric juice (3 h) and intestinal juice (7 h). Different letters (a, b, c) indicate statistically significant differences between groups (*p* < 0.05).

Eventually, three LAB were acquired from two feline milk samples through typical morphological characteristics of LAB, which included Gram-positive, bacilli and cocci, catalase- and coagulase-negative, and nonmotile status, for subsequent assessment. Gram-stain microscope images of three isolates are shown in Figure 1A. The 16S rDNA sequences of the three LAB were detected to confirm the type of these isolates preliminarily. After matching with the existing gene sequences in NCBI GenBank by using Blast, one of three strains was identified as *Weissella confusa* (M1), and the other two strains were identified as *Lactobacillus plantarum* (M2 and M3). The phylogenetic tree of three strains as shown in Figure 1B.

### 3.2. Growth Kinetics

The growth curve of the three isolates within 48 h is shown in Figure 1B. Strain M3 entered the logarithmic phase at the 4th hour, while strains M1 and M2 entered on the 8th hour, and it entered the plateau stage during the 12th hour. The trend of the OD600 value of strain M2 is similar to that of M1, and its leak value also appeared on the 32nd hour, which means that they entered the plateau stage. The highest absorbance values of M1 and M2 were all around 1.9 (1.89 and 1.95, respectively), while the highest absorbance value of M3 was far lower, at only 1.39, showing a lower final growth concentration.

The results of the livability of three isolates in different acidic environments are shown in Figure 1C. Three isolates barely survived in the pH 2 environment, and strain M1 was also unable to survive in the pH 3 and pH 4 environment, while strains M2 and M3 showed a high resistant capacity to the pH 3 and pH 4 environment.

As shown in Figure 1D, all three isolates presented different resistance capacities to a 0.01–0.05% concentration of bile salt. Approximately 80% and 25% of isolates could stay in 0.01% and 0.03% bile salt, and there was no significant difference among the three isolates. When the bile salt concentration rose to 0.05%, the livability of strains M2 and M3 was significantly higher than that of strain M1.

The results of the survival rates of three isolates in simulated GIT conditions are shown in Figure 1E. After dealing with artificial gastric juice for 3 h, all isolates presented high livability, within the range of 58.53% to 95.64%. Regarding tolerating artificial intestinal juice, M1, whose livability is only 10.64%, presented a low survival ability in this simulated environment. The other two strains showed higher tolerance to artificial intestinal juice, whose livability is 92.83% (M2) and 61.75% (M3).

### 3.3. Adhesion Capacity

**Figure 2:**
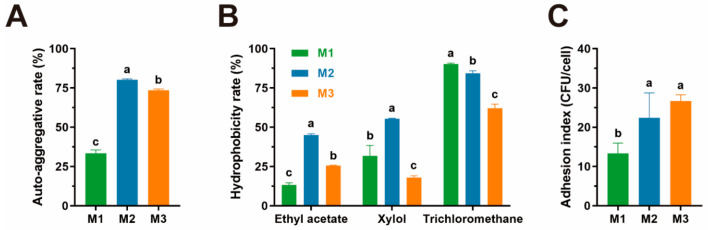
The results of adhesion capacity assessment. All the results are represented as mean ± SD. (**A**) The auto-aggregation abilities of three isolates; (**B**) the cell surface hydrophobicity of three isolates in different organic solutions; (**C**) the adhesion index of isolates to Caco-2 cell lines. Different letters (a, b, c) indicate statistically significant differences between groups (*p* < 0.05).

To assess the adhesion abilities of three isolates, we conducted experiments on the following aspects: auto-aggregation assay, cell surface hydrophobicity assay, and adhesion assay of LAB to the Caco-2 cell line. Strains M1 and M2 show higher auto-aggregation rates compared to strain M1, as shown in Figure 2A.

Regarding cell surface hydrophobicity, the performances of strain M2 in different solutions were better than those of strain M1 and M3, except in trichloromethane, whose cell surface hydrophobicity rate was slightly lower than that of M1, as shown in Figure 2B.

All three isolates presented the ability to adhere to the Caco-2 cell line, and strain M2 and M3 exhibited a greater ability to adhere to cells, whose adhesion index are 22.40 and 26.67, as shown in Figure 2C. Strain M1, however, performs poorly in this part compared to the other two strains.

### 3.4. Safety Assessment

**Figure 3:**
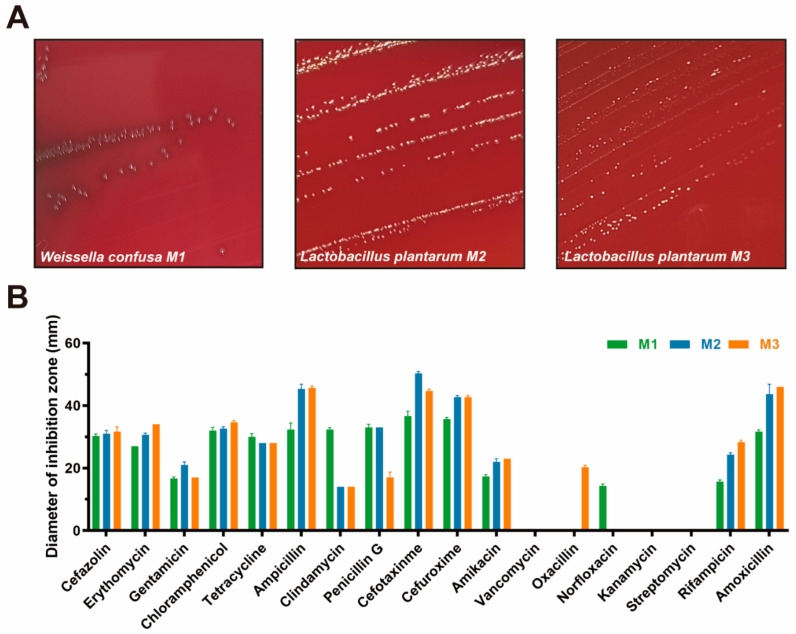
The results of safety assessment. All the results are represented as mean ± SD. (**A**) The hemolysis results of three isolates on the blood plate; (**B**) diameters of different antibiotic inhibition zones of three isolates.

The hemolytic assay results are shown in Figure 3A. Among them, strain M1 presents an γ-hemolytic ring, while the other two strains present an α-hemolytic ring, showing that three isolates are nonhemolytic.

The antibiotic susceptibility of three isolates is shown in Figure 3B and Table 1. We found that strains M2 and M3 presented a similar performance in this assay, and they are resistant to clindamycin, oxacillin, kanamycin, and streptomycin. Strain M1 is resistant to vancomycin, oxacillin, norfloxacin, kanamycin, and streptomycin, showing a weaker antibiotic susceptibility rate (13/18) than strain M3 (14/18).

### 3.5. Antipathogenic Activity

**Figure 4:**
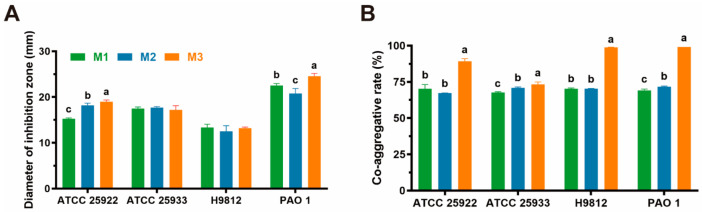
The results of antibacterial-related assay. All the results are represented as mean ± SD. (**A**) Antagonistic activity of three isolates against pathogenic bacteria by the Oxford cup method; (**B**) co-aggregative activity of three isolates against four common gastrointestinal pathogenic bacteria. Different letters (a, b, c) indicate statistically significant differences between groups (*p* < 0.05).

As presented in Table 2 and Figure 4B, all diameters of the inhibition zone of the three isolates are longer than 12 mm, which means that all of them have significant antagonistic activity against four common intestinal pathogens, although their performances are not all the same. It turns out that strain M3 has the best antipathogenic performance, whatever pathogenic bacteria it acts on. Also, three isolates show high antagonistic activity against *P. aeruginosa* (PAO 1). However, compared to confronting the other three pathogens, they all underperformed in confronting *S. braenderup* (H9812), and even so, they had grade II antipathogenic activities.

In the performances of co-aggregative activity, as presented in Figure 3A, all three strains can co-aggregate four pathogens. M3 has the highest co-aggregative abilities against four pathogens among the three isolates, and it also shows extremely strong co-aggregative ability against *S. aureus* (ATCC 25923), *S. braenderup* (H9812), and *P. aeruginosa* (PAO 1).

### 3.6. Antioxidative Capacity

**Figure 5:**
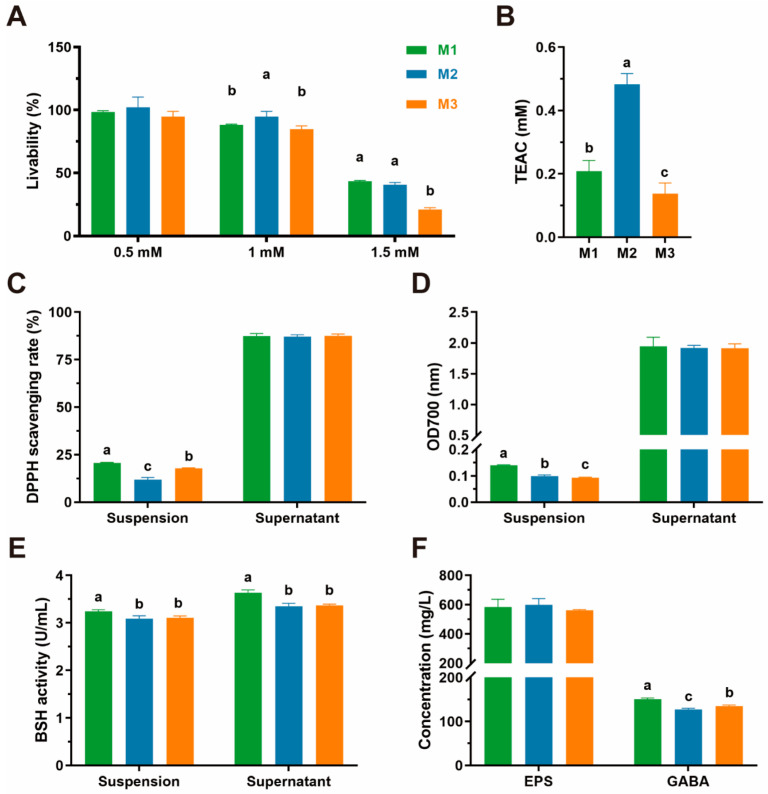
The results of antioxidant ability and probiotic metabolites production evaluation. All the results are represented as mean ± SD. (**A**) Livability of three isolates in different concentrations of H_2_O_2_; (**B**) Trolox-Equivalent Antioxidant Capacity (TEAC) of three isolates; (**C**) DPPH radical scavenging activity of three isolates; (**D**) reducing capacity of three isolates; (**E**) BSH production ability; (**F**) EPS and GABA production ability. Different letters (a, b, c) indicate statistically significant differences between groups (*p* < 0.05).

The antioxidative capacities of three LAB, including tolerance for H_2_O_2_, total antioxidant capacity, DPPH radical scavenging activity, and reducing capacity, are shown in Figure 5A–D, respectively. We find that all three isolates could survive in the different concentrations of H_2_O_2_. The livability of three isolates in 0.5 mM H_2_O_2_, near 100%, showed no significant difference. However, in 1.5 mM H_2_O_2_, the livability of strain M3 was significantly lower than that of strains M1 and M2, at only 21.04%.

Strain M2 exhibited a significantly higher antioxidant capacity (48.30 mM) than strain M1 (20.89 mM) and M3 (13.77 mM).

The DPPH scavenging rates of bacterial suspension range from 20.67% to 11.86%, and strain M1 shows the best level of ability (20.67% ± 0.25). Nevertheless, these numbers are not significantly different in cell-free supernatant.

The reducing capacity results show that there are significant differences among the reducing capacity of the three isolates’ suspensions, which are 0.14 (M1), 0.10 (M2), and 0.09 (M3), respectively. At the same time, no significant difference was observed in the reducing capacity of their supernatants, with values averaging approximately 1.93.

### 3.7. Metabolite Assessment

The results of metabolite assessments of three isolates are shown in Figure 5E,F, which indicate that the EPS production abilities of the three isolates have no significant diversity. However, the GABA and BSH production abilities are significantly different, and strain M1 has the strongest GABA and BSH production ability compared to strains M2 and M3.

## 4. Discussion

The probiotic properties of lactic acid bacteria are species- and strain-specific, which explains why researchers continue to search for novel strains with probiotic potential [28]. Compared to biochemical identification, 16S rDNA sequencing is a more reliable and accurate method for bacterial identification. Therefore, we adopted this method to identify the suspicious probiotics isolated from the feline milk sample. One of them was identified as *Weissella confusa* (M1), and the other two strains were identified as *Lactobacillus plantarum* (M2 and M3), a kind of probiotic that is considered ideal in the food industry [29]. However, genetic analyses need to be more comprehensive in order to compare probiotic bacteria at the strain level [30].

The growth curve primarily reflects the replication capacity and growth kinetics of bacteria, rather than overall metabolic activity. All three isolates presented good growth kinetics, which indicates that these strains could be quickly activated when entering and colonizing the intestine, and maintained a large number of colonies for a long time, which provided a basis for their probiotic effects in the intestinal tract of animals [21]. In order to arrive in the intestine and colonize intestinal cells, probiotics must have the capacity to resist environments with acidic or high levels of bile salt, which are the greatest threats to probiotics in the gastrointestinal tract. In our study, strains M2 and M3 present strong acid and bile salt-resistant abilities, which means that they can resist inclement surroundings and reach the intestine. This result was proved by an assay focused on follow-up tolerance for simulated GIT conditions, whose result displays that strains M2 and M3 retained a high survival rate (92.83% and 61.75%) after dealing with artificial gastric and intestinal juice. After being administrated orally, this high tolerance to simulated GIT conditions helps them arrive in the intestine almost integrally and further reproductive growth.

When isolates arrive in the intestine successfully, the next step is attaching to intestinal epithelial cells. Before conducting cell adhesion assay in vitro, auto-aggregation and hydrophobicity assay can be conducted as precursor experiments to evaluate probiotics’ adhesion abilities preliminarily [31]. Auto-aggregation of probiotics can enrich the gastrointestinal tract to a higher concentration level and promote the formation of biofilm [12,32]. In our study, we found that the auto-aggregation ability of strains M2 and M3 was much higher than that of Kim et al. on probiotics isolated from feline feces, which also explains their strong biofilm formation abilities [33]. However, the poor performance of strain M1 in this test may be related to strain differences, which suggests that the auto-aggregation ability has substantial strain differences. Hydrophobicity can steady the connection of bacteria and intestinal epithelial cells and help bacteria colonize in the intestine successfully [34]. Compared to the previous study about LAB isolated from pig feces published by Sirichokchatchawan et al., our three isolates showed lower hydrophobicity to xylol [35]. To further evaluate probiotics’ adherence to cells in real situations, we conducted the assay of adhesion to the Caco-2 Cell Line, which is frequently applied to build an intestinal interaction model, and the result shows that three isolates have a 13.3–26.8 adhesion index to it [20]. Among them, strains M2 and M3 presented higher Caco-2 Cell Line adhesion ability, which was consistent with the results of auto-aggregation and hydrophobicity assays.

Although LAB are generally considered safe for animals, certain strains may have potential pitfalls, which needed be eliminated to ensure no risk at all in clinical applications [36]. All the potential probiotics must be proven to be nonhemolytic first to ensure they can be applied in vivo, and our result showed that three isolates preliminarily met this condition in sheep blood plates. Susceptibility to an array of commonly used human and veterinary antibiotics should be assessed for all potential probiotic strains. This procedure is essential in detecting potential probiotic strains with transferable antibiotic-resistance genes, which may be detrimental to the host [12]. All three isolates are susceptible to KZ, E, GM, C, TE, AMP, P, CTX, CXM, AK, RD, and AML and resist VA, NOR, KZ, and S. In the literature, strains of lactobacilli are generally resistant to β-lactam antibiotics (including Penicillin, Ceftriaxone, Ampicillin, and Oxacillin) as a result of the presence of β-lactamase in such strains [37]. However, our results are almost contrary to this conclusion, which may be induced by some factors like the source and geographical locations of probiotics [38]. It also further proves that the susceptibility to antibiotics of probiotics is species- and strain-specific [39]. Concerning their antibiotic resistance, the isolates of the present study showed different levels of resistance in previous research, which reported that LAB always has aminoglycoside (gentamicin and streptomycin) and glycopeptide (vancomycin) resistance because their innate resistance resulting from the impermeability of their membrane, presumably through a resistance efflux mechanism [12]. Although antibiotic-resistant bacteria have led to conventional treatment of bacterial infections becoming increasingly ineffective and may be a potential threat to public health, the intrinsic resistance of probiotic strains promotes both therapeutic and preventive benefits when administered together with antibiotics, as intestinal microbiota [37]. In conclusion, all three isolates presented high-security in vitro tests, while genetic and in vivo tests should be conducted to confirm it further.

The antimicrobial abilities of probiotics, especially in today’s epidemic of drug-resistant bacteria, are critical and valuable in treating infections caused by drug-resistant bacteria. All the examined strains were able to antagonize the growth of four standard common pathogenic strains, which is consistent with a previous study which reported that LAB has broad spectrum of antagonistic activity against both Gram-negative and Gram-positive pathogens [12]. In particular, they had better inhibitory effects on *E. coli*, *S. braenderup*, and *P. aeruginosa*, which is consistent with Weissella strains isolated from horse feces and Lactobacillus strains reported by previous studies [28,40]. At the same time, strains M2 and M3 presented a longer inhibition diameter to *E. coli* and *S. aureus* compared to Weissella strains isolated from horse feces, which means that they can be potential candidates against these two pathogens [40]. Lactic acid bacteria always present their antimicrobial activity by secreting various organic metabolites like organic acid (always lactic acid), bacteriocins, and hydrogen peroxide [41]. Interestingly, a great deal of research has reported that exopolysaccharides (EPSs), which we evaluated in our study, also have antimicrobial activity and could be potential antimicrobial agents [42]. Therefore, lactic acid bacteria exerting antimicrobial function are involved in a mass of complicated mechanisms concerned with a series of metabolite interactions [43]. But this is puzzling and represents a future research direction at the same time. In my opinion, illuminating how a specific strain plays an antibacterial role systematically and scientifically based on previous studies is worth further study and discussion. Co-aggregation is one of the desired properties of probiotics. It may play an essential role in eliminating pathogens in the gastrointestinal tract by preventing adherence of pathogens to the host tissue. Thus, the co-aggregation abilities of probiotics act on behalf of the antimicrobial abilities, in part. In this study, the results of antimicrobial ability and co-aggregation ability were basically consistent, which confirmed this point. And compared to 14 strains of LAB reported by Zhang et al. [15], strain M3 presented outstanding co-aggregation capacity against all four pathogens. To sum up, strain M3 presented excellent antibacterial ability and had in vivo antimicrobial research value.

In recent years, as more and more probiotic research is ongoing, except for antibacterial capacity, many more probiotic functions have been excavated, including antioxidative and beneficial metabolite production functions [21,33]. In addition, the antioxidative and beneficial metabolite production function may have an underlying connection with the antibacterial function. That is why we evaluated three feline milk source isolates’ relevant capacity for the first time, and we hope to provide theoretical references for subsequent studies in this way.

During the occurrence of oxidative stress, reactive oxygen species (ROS) such as H_2_O_2_, DPPH, OH^−^ and O_2_^−^ will be generated rapidly in large amounts. Moreover, their unrestrained accumulation may induce a series of diseases to occur and develop, like inflammation, cancer, atherosclerosis, ageing, as well as degenerative disease [44]. Various research studies have reported that probiotics can scavenge ROS and act as antioxidants, and we evaluated the antioxidative capacity of isolated probiotics from this perspective. Our isolates are almost unaffected by H_2_O_2_ at low to medium concentrations, maintaining a survival rate of nearly 100%. The result of the total antioxidative capacity assay showed that strain M3 has the strongest antioxidative capacity (amount to 0.48 mM Trolox), which was higher than 13/16 strains of LAB reported by Kim et al. [33]. Moreover, their supernatants’ abilities of DPPH radical scavenging (87.15–87.53%) are much higher than the reports (4.70–53.55%) by Kim and Zhao et al. [17,33]. The results above make it abundantly clear that the three isolates all showed excellent antioxidative capacity and had the potential to develop into antioxidant agents.

Exopolysaccharides (EPSs), γ-aminobutyric acid (GABA), and bile salt hydrolase (BSH) are three kinds of common bacterial metabolites with various probiotic functions. Among them, EPSs are extracellular biopolymers derived from LAB with antioxidant, immune-regulating, maintain microbiota homeostasis, and other biological activities [45]. GABA is a kind of inhibitory neurotransmitter in the central nervous system of mammals. Besides this fundamental function, it is also able to activate immunocytes, inhibit cancer cell growth, stimulate appetite, and have lots of beneficial effects [46]. BSH eliminates the adverse effects of bile salts by hydrolyzing bile salts, which play a role in providing energy for intestinal microorganisms, improving cell vitality, enhancing antibacterial ability, and maintaining the balance of intestinal microbiota [21]. The previous research reports that the EPS production abilities of the other sources of LAB range from 20 to 370 mg/L, and three isolates in this study are above 500 mg/L (560.86–598.84 mg/L), which indicates they all have strong EPS production abilities [47]. In the present study, however, the GABA production abilities of three LAB (0.13–0.15 g/L) are slightly weaker than those of L. plantarum 8014 (0.16 g/L) reported by Li et al. [48]. Tsai et al. [49] reported that only 22 strains displayed BSH activity among 800 strains of LAB. Compared with these strains’ poor performances, our isolates all displayed BSH activity, which is consistent with the result reported by Wang et al. [21]. This study evaluated the beneficial metabolites of feline potential probiotic strains for the first time, which can provide valuable reference significance for subsequent studies. Moreover, our results showed that compared with other derived probiotics, feline potential probiotic strains can produce more probiotic metabolites with beneficial functions and present excellent probiotic properties.

## 5. Conclusions

In conclusion, our study isolated and evaluated three strains of LAB isolated from feline milk from multiple dimensions. We found that *Lactobacillus plantarum* M3 has good performance in terms of growth, resistance, adhesion, and antioxidative properties, which means that it could serve as supporting therapy or in combination with antibiotics to treat multidrug-resistant bacteria infections. There are, however, still many problems to be solved on the road to apply clinically, such as genetic level and in vivo evaluation. Otherwise, we evaluate three feline milk source isolates’ antioxidative and beneficial metabolite production capacity for the first time, and we hope to provide theoretical references for subsequent studies in this way.

## Figures and Tables

**Table 1:** Antibiotic susceptibility of three LAB isolated from feline milk to different antibiotics.

Strain	Antibiotic Susceptibility																		Sensitive Rate (S + I, %)
	KZ	E	GM	C	TE	AMP	DA	P	CTX	CXM	AK	VA	OX	NOR	KM	S	RD	AML	
M1	S	S	I	S	S	S	S	S	S	S	I	R	R	R	R	R	I	S	72.22
M2	S	S	S	S	S	S	R	S	S	S	S	S	R	R	R	R	S	S	72.22
M3	S	S	I	S	S	S	R	I	S	S	S	S	R	I	R	R	S	S	77.78

KZ: cefazolin, E: erythromycin, GM: gentamicin, C: chloramphenicol, TE: tetracycline, AMP: ampicillin, DA: clindamycin, P: penicillin G, CTX: cefotaxime, CXM: cefuroxime, AK: amikacin, VA: vancomycin, OX: oxacillin, NOR: norfloxacin, KM: kanamycin, S: streptomycin, RD: rifampicin, AML: amoxicillin. Antibiotic susceptibility was classified as resistant (R), moderately susceptible (M), or sensitive (S) based on zone diameter (mm), following CLSI guidelines.

**Table 2:** Antagonistic activity of three isolates against pathogenic bacteria by the Oxford cup method.

Strain	Diameter of Inhibition Zone (mm)			
	*E. coli* ATCC 25922	*Staphylococcus aureus* ATCC 25923	*Salmonella* H9812	*P. aeruginosa* PAO 1
M1	15.27 ± 0.16 II	17.62 ± 0.27 III	12.99 ± 0.27 II	22.49 ± 0.45 IV
M2	18.25 ± 0.46 III	17.64 ± 0.15 III	12.32 ± 1.17 II	21.12 ± 0.90 IV
M3	19.20 ± 0.16 III	17.65 ± 0.50 III	13.29 ± 0.19 II	24.31 ± 0.45 IV

Antipathogenic activity was classified as level II (12–16 mm), III (16–20 mm), and IV (>20 mm), according to zone diameter as described in a previous study.

## Data Availability

The datasets used and analyzed during the current study are available from the corresponding author upon reasonable request.
